# A Novel Mixed Methods Approach to Synthesize EDA Data with Behavioral Data to Gain Educational Insight

**DOI:** 10.3390/s20236857

**Published:** 2020-11-30

**Authors:** Clodagh Reid, Conor Keighrey, Niall Murray, Rónán Dunbar, Jeffrey Buckley

**Affiliations:** 1 Faculty of Engineering and Informatics, Athlone Institute of Technology, Athlone N37 HD68, Ireland; c.keighrey@research.ait.ie (C.K.); nmurray@ait.ie (N.M.); rdunbar@ait.ie (R.D.); jbuckley@ait.ie (J.B.); 2 Department of Learning, KTH Royal Institute of Technology, 114 28 Stockholm, Sweden

**Keywords:** electrodermal activity, wearables, cognitive load, education, behavior

## Abstract

Whilst investigating student performance in design and arithmetic tasks, as well as during exams, electrodermal activity (EDA)-based sensors have been used in attempts to understand cognitive function and cognitive load. Limitations in the employed approaches include lack of capacity to mark events in the data, and to explain other variables relating to performance outcomes. This paper aims to address these limitations, and to support the utility of wearable EDA sensor technology in educational research settings. These aims are achieved through use of a bespoke time mapping software which identifies key events during task performance and by taking a novel approach to synthesizing EDA data from a qualitative behavioral perspective. A convergent mixed method design is presented whereby the associated implementation follows a two-phase approach. The first phase involves the collection of the required EDA and behavioral data. Phase two outlines a mixed method analysis with two approaches of synthesizing the EDA data with behavioral analyses. There is an optional third phase, which would involve the sequential collection of any additional data to support contextualizing or interpreting the EDA and behavioral data. The inclusion of this phase would turn the method into a complex sequential mixed method design. Through application of the convergent or complex sequential mixed method, valuable insight can be gained into the complexities of individual learning experiences and support clearer inferences being made on the factors relating to performance. These inferences can be used to inform task design and contribute to the improvement of the teaching and learning experience.

## 1. Introduction

Electrodermal activity (EDA), also referred to as galvanic skin response (GSR), relates to electrical changes that occur in the skin [1,2,3,4,5,6,7,8]. EDA measurement gauges psychophysiological activity of the sympathetic nervous system which is a part of the autonomic nervous system [3,9]. Sweating is normally associated with thermoregulation of the body, however, in response to psychological stimuli the body produces the physiological response of sweat through plantar and palmar sites [8]. This sweating causes an increase in the electrical conductance of the skin as part of the autonomic response [10], leading to EDA being employed for evaluating autonomic function and assessing levels of cognitive or emotional reaction to an arousing event [1,2,3,4,5,6,7,8]. Measuring EDA using wrist-worn sensors provides an unobtrusive implicit indicator of these reactions experienced through engagement with a task [8,9,11]. EDA measurement affords the capacity to gain understanding of how an individual experiences an event without restricted movement where the authenticity of the activity is minimally affected. This has proven valuable in a number of disciplines including but not limited to biomedical engineering [11], neuroscience [12], behavioral science [13] and education [14]. In education research, the use and interpretation of EDA data has been conducted exclusively through quantitative approaches. This article discusses the value that EDA data can provide via a mixed method mode of inquiry.

In the context of education, Thammasan et al. [1] have demonstrated the feasibility of monitoring EDA signals in educational settings. EDA measurement has been used to investigate performance during examinations and design activities with specific focus on cognitive function and response [14,15]. It has also been used in the identification of talented students [16] and in measuring cognitive load during arithmetic and reading tasks [17]. As monitoring of EDA signals is feasible in educational settings, it is timely to consider the insights that they can provide. This work aims to advance the utility of monitoring EDA in education through presenting a novel method for the synthesis of EDA data with behavioral data to gain insights into learners’ educational experiences.

To date, EDA research in education has focused on quantitative measurement and interpretation. Previous works have evaluated measures of EDA via statistical means, with examples of such approaches including correlation analyses, t-tests between datasets, multiple regression modelling and event marking. Although such approaches address the fundamental research questions of existing works, limitations were outlined by the authors including a lack of capacity to mark specific events in the data and to explain other variables impacting on performance outcomes [14,15]. From the solely numerical data output of EDA, there is no capacity to explain variables impacting on performance. These limitations are addressed through the following aim of this paper.

The primary aim of this paper is to offer perspectives on how to advance the utility of physiological sensors in educational research by detailing a novel two-phase convergent mixed methods approach where quantitative EDA data are synthesized with qualitative behavioral data to obtain more in-depth interpretations of a learner’s experiences. The overall method presented also includes an optional third sequential explanatory phase, which changes the method into a complex mixed methods approach [18]. The outlined novel convergent mixed method aims to support in-depth analysis of cognitive load experienced during problem solving in educational settings. It is intended that this approach could be used as a means of advancing insight into learner performance and inform curriculum design, specific task design and educational practice. 

## 2. Research Context and Methodological Requirements

### 2.1. Cognitive Load in Education

“Cognitive load theory aims to explain how the information processing load induced by learning tasks can affect students’ ability to process new information and to construct knowledge in long-term memory” [19] (p. 261). The premise of the theory is that individuals’ limited working memory capacity, their capacity to temporarily hold and process information, can constrain cognitive processing [19,20]. Cognitive load is a cognitive reaction that is increased when demands are placed on the cognitive system. When this load becomes too high, it can hinder an individual’s capacity to learn and their motivation to engage in similar situations in the future [19,20,21,22]. This load can be increased through insufficient instructional methods and unnecessary distractions [19]. The goal of cognitive load theory is for innovative and effective instructional procedures to be generated to manage the load imposed on working memory and optimize information processing capacity [20]. In line with this goal and to support learning, it is important that thorough investigations are conducted to provide detailed insight into the effect of cognitive load on performance and to identify elements of educational tasks that cause undesirable increases in cognitive load so that the potential for learning can be optimized. Measures of cognitive load can be broadly divided into two categories, subjective or objective. According to Brünken et al. [23], subjective measures of cognitive load include self-reported invested mental effort (indirect) and self-reported difficulty of materials (direct), where objective measures include psychophysiological measures such as pupillometry or EDA (indirect), and brain activity and dual-task performance (direct). Table 1 outlines the classification of these approaches to measuring cognitive load. 

In order to situate the synthesis of EDA and behavioral data relative to the measurement of cognitive load, the following sections provide a brief overview of some subjective and objective cognitive load measures and their advantages and disadvantages.

#### 2.1.1. Subjective Measures

Subjective rating scales can be used to determine an individual’s level of agreement with a statement or intensity of a feeling or emotion in response to an event [24]. Examples include self-reporting Likert scales and semantic differential scales which have been commonly used to measure cognitive load [22,25,26,27,28,29]. The number of points on the scale can vary. In certain approaches, seven-point scales such as the NASA Task Load Index (NASA-TLX) are implemented whilst others employ nine-point rating scales in cognitive load measurement [30]. NASA-TLX was developed with the goal of providing a sensitive summary of variations of workload [31]. The rationale and process of development of the scale are documented by Hart and Staveland [31]. NASA-TLX is a direct subjective measure of cognitive load [25]. Paas [32] developed and validated a nine-point Likert-type item to evaluate the mental effort experienced by an individual as they performed a task. The numbers on the scale were assigned labels ranging from (1) very, very low mental effort to (9) very, very high mental effort [32]. The difference between the two scales, apart from the number of points, is that the scale developed by Paas [32] is a single item that solely measures mental effort. The NASA-TLX has multiple items that measure various factors which contribute to workload such as mental demand, effort and frustration [31]. Each approach offers a valid and reliable subjective measure of cognitive load [22]. The scales can be administered multiple times throughout an activity or once at the end of a series of activities [27]. Research findings have indicated that a single retrospective measure yields a higher response than the average of the multiple measures after each activity [27]. Rating scales, however, have some limitations. There is no assumption of equal intervals between each of the ratings. There is a tendency on five-point and seven-point scales for individuals to avoid selecting extreme values on the scale, and there is no way of knowing if the individual completing it wished to add a comment on what was being investigated [24]. It is also noted that frequent administration throughout a learning experience can be intrusive [25]. However, some of these issues can be addressed through method design, e.g., using a nine-point scale or adapting the standard format to include a comment section.

There are various subjective qualitative approaches suitable to support investigations of cognitive load and for gaining understanding and insight of experiences from individual perspectives. These include interviews such as stimulated recall interviews [33] (verbal data) and concurrent verbal protocols such as think-aloud [34]. Each of these approaches have both strengths and weaknesses associated with their application. Stimulated recall interviews have been used in cognitive load research to gain an insight of thought processes and how they relate to different types of load experienced [33]. Cohen et al. [24] and Creswell and Creswell [18] discuss in detail, the advantages and disadvantages of interview approaches. They outline that interview approaches, not specifically for the purpose of cognitive load measurement, can be used to gather information directly relating to research questions or objectives, to test or generate hypotheses, or in conjunction with other methods to examine and validate the methods or investigate the motivations and responses of individuals. However, some weaknesses associated with interview approaches exist such as interviewer bias or interviewees becoming uneasy with a line of questioning. 

Think-aloud protocols provide a method for studying both behavioral and cognitive processes during problem solving [35]. These protocols have been used with implicit measures of cognitive load such as eye tracking to inform a detailed account of performance and performance parameters [33,35]. However, despite these protocols informing the approach being implemented in real-time, there are some weaknesses to their implementation such as slowing participants down, which in turn makes tasks take longer and changes the participant’s interaction with a task [35,36]. In addition, when used simultaneously with eye-tracking measurement it can lead to an increased number of fixations [35].

In the context of this work, the selection of a subjective measurement method must primarily be based on its capacity to reliably measure cognitive load and capacity to compliment the objective measurement employed for triangulation.

#### 2.1.2. Objective Measures

As detailed in Table 1, behavioral measures and physiological responses are examples of objective measurement of cognitive load. Insights to behavior can be gained subjectively through approaches such as think-aloud protocols [35], or objectively using approaches such as observation. Observations provide capacity for the researcher to capture situations such as events and behaviors as they occur and afford strong face validity through capturing rich contextual information [18,24]. Limitations in their use include the researcher being seen as intrusive and there may be problems in gaining rapport with certain participants [18]. Video recording can also be used as a means of observation to circumvent limitations in building rapport. In video observations, the collection of footage by an observer may be disruptive to the participant or affect responses [18]. However, recording equipment can be discretely setup to minimize intrusiveness. Using video also affords capacity to observe behaviors retrospectively. Audio–visual recordings can be used to support additional data collection through interview techniques, such as video-stimulated recall interviews, to provide an in-depth understanding of events [37].

The measurement of physiological responses as an indicator of cognitive load is based on the premise that changes in cognitive load are reflected by physiological variables [22]. Various physiological responses have been used as objective, but indirect, measures to investigate cognitive load experienced by individuals throughout activities. These include eye-tracking, pupillometry, electroencephalography (EEG), heart rate (HR) and EDA [3,22,23,38]. In using physiological responses as measures of cognitive load, it is necessary to observe additional variables to triangulate the measurement to evaluate whether it can be interpreted as an indication of cognitive load [25]. Physiological measures afford an objective measure of cognitive load during the completion of a task. However, they often require the use of invasive technologies, which themselves have been criticized due to the potential negative impact that they can have on primary task performance and therefore the ecological validity of a study [22,29]. However, recent advances in technology have provided capacity to measure physiological responses such as HR and EDA unobtrusively, e.g., wearable wristbands [8,9,11]. This increases the viability of implicitly measuring cognitive load through engagement with a task, as significant movement restrictions are no longer a concern.

The most important factor to consider is whether these physiological measures can be validly interpreted to measure cognitive load. The validity of HR and HR variability to measure cognitive load is contested. Paas and van Merriënboer [39] detailed these measures as invalid and insensitive to slight fluctuations in cognitive load following a spectral-analysis technique of HR variability. Solhjoo et al. [40], however, in conducting a correlation analysis between HR and HR variability and self-report measures of cognitive load reported a strong positive correlation between these indirect measures of cognitive load and HR variability. However, it is important to note the small sample size (n = 10) included in that study.

As mentioned, EDA measurement relates to changes in the skin in response to an event [1,2,3,4,5,6,7,8] where it has been used in the implicit measurement of both cognitive and emotional reactions [3,4,5,11,12,13,14,39]. EDA signals can vary between two categories, tonic change or phasic change [7]. The phasic component is referred to as skin conductance response (SCR) and is associated with short increases in EDA caused by arousing events such as sound, sight or smell [1,7]. The tonic component is referred to as skin conductance level (SCL) and is associated with slow change in skin conductance [1,3,7]. These changes can be caused by an increase in cognitive activity [3,7]. Thammasan et al. [1] detail the process and importance of differentiating between the tonic and phasic components in the analysis of EDA data.

In summary, the selection of a method of cognitive load measurement should be based on the evaluation of its appropriateness to the subject under investigation [41]. In the context of this work, to present a convergent mixed method to investigate cognitive load during performance on a task, it is necessary that an individual’s movements would not be limited. From the perspective of objective measurement of cognitive load, EDA would provide a suitable objective measure as it can be measured using unobtrusive wrist-worn physiological sensors [8,9,11]. Simultaneously collecting behavioral observations through video affords capacity to gain a further insight into the EDA data not previously achieved. It would also increase the richness of the data and support further explanation of variations in EDA and behaviors. A subjective measure, such as the self-report scale developed by Paas [32], would be suitable to both measure cognitive load and triangulate with EDA and behavioral data as the study seeks to examine cognitive load and not separate elements of workload which the NASA-TLX would provide [31].

This paper offers further potential to the largely quantitative methods demonstrated in measuring EDA in educational environments by including an additional qualitative perspective to form a novel mixed methods synthesis of behavioral and EDA data.

## 3. The Mixed Methods Approach

EDA sensor data are typically analyzed in a quantitative manner [3,8,10,41]. When EDA measurement is used in isolation there is no capacity for the numerical output to explain why any changes occurred. Explaining why a change or reaction occurred requires supplementary and/or additional data collection for triangulation [25]. 

Graphing EDA data in relation to performance over time affords the capacity for interpretation and exploration of the causes of EDA fluctuations, and therefore changes in cognitive load, during a task. This interpretation and exploration can include using behavioral analysis to explain changes in EDA and to explore how fluctuations in EDA might manifest into behaviors. To conduct this form of detailed inquiry, explanatory qualitative methods are required to explain periods of interest in the data and provide detailed insights into the experience of individuals when engaging with a task. Combining the EDA data as an indirect measure of cognitive load with complementary subjective measures and subsequent qualitative analysis can lead to an in-depth investigation of the cognitive load experienced by an individual during a task and add to the validity of causal inferences.

**Figure 1:**
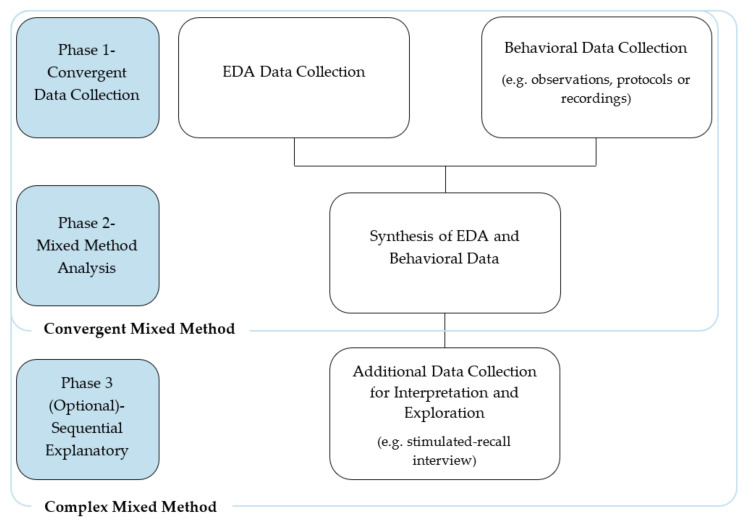
Method description.

In the context of the work presented, to address limitations in existing approaches using EDA measurement in education and expanding the utility of physiological sensors in educational settings, the proposed convergent mixed method design can be considered in the initial two phases depicted in Figure 1. In phase one, EDA and behavioral data are simultaneously collected. Phase two involves the synthesis of the EDA and behavioral data. The optional phase three would act as an additional sequential explanatory phase and focus on the collection of complementary data to contextualize and explain the timepoints of interest identified in phase two. This additional phase, if included, changes the overall method to a complex sequential mixed methods approach [18].

### 3.1. Phase 1—Convergent Data Collection

The convergent mixed method parallel design has both qualitative and quantitative data that are collected independently and in parallel to each other [24]. These data converge and yield triangulation which offers complementary data on the phenomenon in question [24,42]. In the context of this work, it refers to the collection of behavioral observations and EDA data in parallel for insights into the effect of cognitive load on educational performance.

#### 3.1.1. Observing Behaviors

Observation of behavior can include memo writing by a facilitator while visually observing, using protocols such as think-aloud, or using imagery or audio–visual recordings to support retrospective observations [18,24]. In using observations, capacity is provided to capture situations such as behaviors or important events as they occur relative to each individual. This would be similar to the approach employed by Cain and Lee [9], whereby images afforded the capacity to identify engagements in a maker space that cause arousal across participants and engagements that caused arousal in certain individuals. Observations afford strong face validity through capturing rich contextual information [18,24]. Capturing this additional data also affords the capacity to triangulate measurements [25]. There are advantages and limitations to observational approaches, as earlier outlined. The selection of an observational method is ultimately dependent on the requirements of each individual study. In the context of this work, collection of video observations affords the capacity to retrospectively observe behaviors exhibited during a task and facilitate further sequential explanatory investigation with approaches such as video-stimulated recall interviews. Recording equipment should be set up discretely to minimize intrusiveness and protocols put in place to ensure ethical treatment and storage of the data.

#### 3.1.2. Collecting EDA Data

EDA is measured in micro Siemens (μS) and the rate that it is sampled at depends on the device being used to collect the measurement. The Empatica E4, for example, samples EDA at 4 Hz [6] while the Pip biosensor samples EDA at 8 Hz [7]. EDA data can be collected during performance on any type of task depending on the requirements of the investigation. The collection of EDA data can be complemented by using appropriate subjective measures of cognitive load, such as Likert scales, depending on the qualitative judgement of the investigator. For a comprehensive account of collecting EDA data through wearable sensors, see Braithwaite et al. and Villanueva et al. [41,43].

In measuring physiological responses such as EDA, it is essential that baseline measurements are obtained so that the EDA data during the task can be compared to an individual’s EDA when they are in a relaxed state [3]. To obtain the baseline measurement, the sensor should be worn on an individual’s non-dominant wrist with no movement for a specified period of time for calibration and collection of the baseline data [44]. The recommended period of time for collecting baseline data varies from 2 to 20 minutes across various studies and reports [3,7,41,45]. In their report on analyzing EDA data, Braithwaite et al. [42] recommend a period for baseline measurement between 2 and 4 minutes for quantitative analysis of the data, while Keighrey et al. [7] propose 5 minutes and Villanueva et al. [44] state between 5 and 10 minutes.

Following the baseline period, the EDA of the individual is collected as they engage with a task or problem. Some sensors that capture EDA automatically timestamp the data when the sensor is connected to a computer for upload of the information. In this process the time is synchronized with the operating system time data on the computer that the sensor is connected to. While this provides the time that the measurement begins and finishes, it is important for the convergent method being proposed, that the times of key events are also noted so that insights can be gained into performance. The syncing of this critical data is discussed later in this section. An example of a key event may be when an individual progresses to the next element of a task or problem or interacts with a task in a way that is of interest to the study in question.

**Figure 2:**
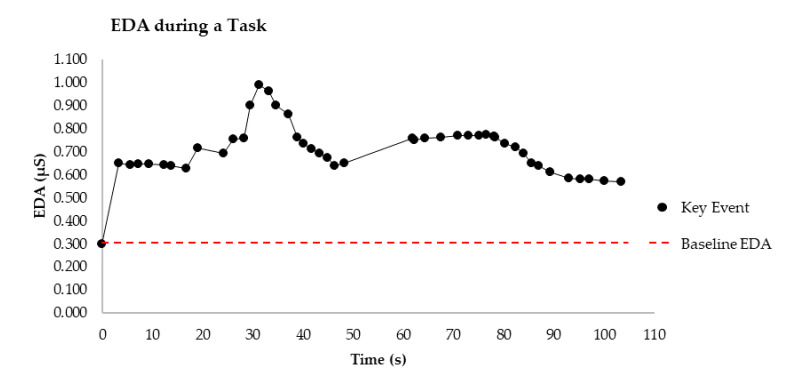
Electrodermal activity (EDA) variation from baseline at key events.

For this method, a bespoke time and movement mapping software was developed to track relevant interactions as users progressed through the completion of the task at hand. This addresses the lack of capacity in existing approaches using EDA in education to mark specific events in the data, highlighted by Villanueva et al. [14]. The software records the period during which participant’s baseline EDA measurements are obtained and has the capacity to note key moves/interactions through monitoring and input by the investigator as the individual engages with the task. A visualization of the outputted data after they have been cleaned can be seen in Figure 2. The cleaning of EDA data is used to remove noise in the signal caused by either internal or external factors [45]. Each of the points on the graph represent a key event, the time the event took place and recorded EDA at that time. A critical element of this method is that the time and movement mapping software is running on the same device that the EDA sensor is connected to. In doing this, the timestamps of key events are synchronized with the operating system and sensor time data. This means that the time of the key events can be accurately mapped onto the EDA data as the time of occurrence is captured by the same device. It is this approach that enables the convergence of the collected data.

### 3.2. Phase 2—Mixed Method Analysis

Traditionally, methods of processing EDA would be followed by a statistical analysis at this point. This method initially involves graphing the cleaned EDA data for synthesis with qualitative behavioral data to contribute to the valuable insights that this approach can provide. The data can be graphed (as illustrated in Figure 2) providing a clear visual overview of the entire experience which can support explanation of other variables impacting on performance outcomes. Subsequent qualitative coding of the observed behaviors collected in Phase 1 can be used to examine EDA trends allowing for further inquiry into associated behaviors. Examples of potential means of inquiry are presented below.

#### Graphical Interpretation of EDA Data

**Figure 3:**
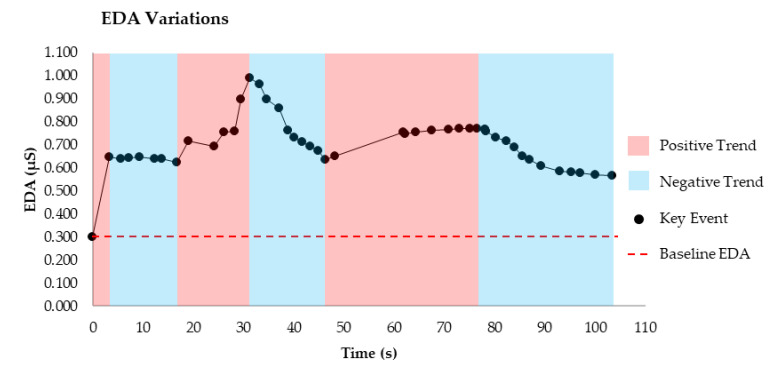
Exploring increases and decreases in EDA in the data.

Recording progress with the problem and the time that it occurs affords the capacity to identify periods of the problem where increases or decreases in EDA occurred. Figure 3 illustrates an example of how these periods might be visually explored relative to the data. The red areas illustrate a positive trend in EDA and blue areas illustrate a negative trend in EDA, similar to the arousing and unarousing engagements explored by Cain and Lee [9]. In this instance, a trend is identified as three consecutive points in a particular direction. Within this approach, the trends in the EDA data are identified, and the behaviors causing the variations subsequently examined. In the context of cognitive load, if there was a specific element of the problem that caused a long delay (relative to the duration of the individuals’ other moves) and adverse increases in cognitive load for several individuals solving the problem at that same point, factors such as instructional design of the problem might be considered to address this. For further insights of the effects of instructional design on cognitive load, see Sweller et al. [19]. This is not to say that all periods where progress is not visibly being made are adverse delays or are directly caused by instruction or presentation of the problem. These periods can also represent where thinking and decision making are taking place.

Qualitative coding of the observed behaviors represents another potential method of synthesizing the EDA data and factors that cause fluctuations in activity during problem solving. Through this approach, the behaviors are coded first and then mapped onto the EDA data. There are several approaches that can be considered for coding the data such as descriptive or process coding or theming the data [46], depending on the focus of each individual study. The coding process will be based on the qualitative judgement of the investigators. Saldaña [46] provides an overview of different considerations for approaches to coding different types of data. The complexity of qualitative inquiry requires rigorous and methodical approaches for valid and trustworthy results [47]. For other researchers to evaluate the trustworthiness of the coding process, it is important to be clear about what was done, why it was done and provide a description of the analysis methods [44,46]. As with assessment criteria in quantitative research for validity and reliability, there are criteria for trustworthiness. These criteria include credibility, transferability, dependability, and confirmability [47]. Nowell et al. [47] outline an exemplar study for achieving these criteria in a thematic analysis. Triangulation (of both data collection and researcher), documenting of the process, peer debriefing, an audit trail and rationale for theoretical, methodological and analytical decisions made are core components of achieving the criteria for trustworthiness [47]. Therefore, it is imperative that the entire coding process is documented in detail at all stages for transparency and clarity around the decision-making process.

**Figure 4:**
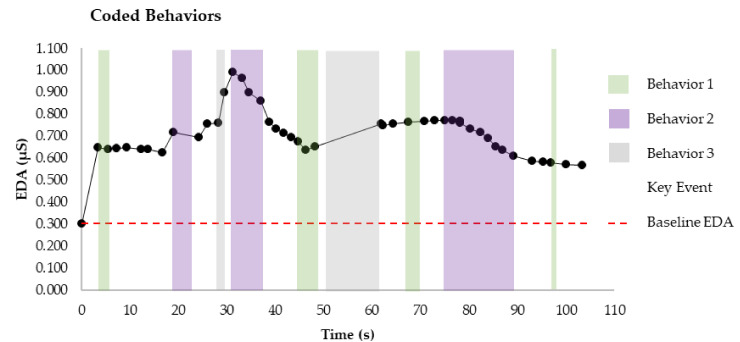
Exploring behaviors of individuals relative to EDA.

With the use of the collected video observations, the behavioral codes associated with solving the problem can be produced retrospectively. The time in the problem-solving process when each of these behaviors are exhibited by the individual can be coded and mapped on to the EDA data, as illustrated in Figure 4. These codes could also be considered with respect to other physiological responses that a sensor might gather along with EDA, such as HR. Mapping the behavioral codes on to the EDA data can support further exploration and investigation of the impact of these behaviors on cognitive load experienced during problem solving. Viewing the coded data from a positivist perspective, the frequency of occurrence of behaviors might be considered [48]. In this regard, the frequency of occurrence of categories of behaviors could be investigated to determine whether the occurrence of certain categories might be associated with increases or decreases in EDA, i.e., if certain categories of behavior are associated with increases or decreases in cognitive load. Should an association be identified, in observing problem solving in the same context in future, these observable behaviors could be extrapolated as an indication of an increase or decrease in cognitive load. In viewing the data from an interpretivist perspective, one might investigate the reason for the behavior from the viewpoint of the individual being observed [48]. To conduct this form of investigation, additional data collection becomes critical to gain insights from the perspective of the subject being observed.

### 3.3. Phase 3 (Optional)—Sequential Explanatory Data Collection

The selection of an appropriate complementary method(s) to collect data to contextualize and explain key phases and delays is ultimately reliant on the aim, questions and variables being considered in a specific study. Questions that might be considered depending on the perspective being taken include: Why is EDA increasing at a specific stage of a problem for several individuals? What behaviors are influencing EDA and why these particular behaviors? Are there any additional effects on the individual and are these positive or negative? How did the problem solver feel about the experience? The questions to be addressed from the data will again be a qualitative judgement made by the individual implementing the method in the context of their own work. As previously indicated, there are a broad range of options available to obtain these additional insights. Some examples of these include interviews, video and concurrent verbal protocols [18,24].

The use of audio–visual equipment to record an individual’s engagement with a problem when EDA is being measured, as outlined in this method, can support understanding of the psychological meaning of fluctuations in the data when it is being analyzed [10]. These recordings can be coupled with interview approaches, such as video-stimulated recall interviews, which can contribute to an in-depth understanding of events of interest from the perspective of the problem solver and produce meaningful explanations [37]. With the presented mixed methods approach, video-stimulated recall interviews would provide suitable complementary perspectives to the qualitative inferences that can be made from the EDA data. The utilization of video-stimulated recall interviews would also be suitable as it would afford capacity to focus on critical timepoints of interest in the EDA and behavioral data, such as trends or delays, while providing the capacity to ask open-ended questions to afford participants the opportunity to express their own views on the situation. This additional phase would change the approach from a convergent mixed method to a complex mixed methods approach.

The collection of complementary data is essential in gaining insight into the problem-solving experience from the perspective of the problem solver. While the quantitative EDA data can be synthesized with the qualitative behavioral data, the addition of interviews or video-stimulated recall interviews in the context of this method affords capacity to move beyond assumptions and speculations of what is happening so that inferences can be made around performance factors in problem solving in education.

## 4. Added Research Value and Conclusions

This work aims to address limitations in existing approaches to using EDA to evaluate cognitive load in educational settings and to advance the utility and remit of sensor technology in education research. Existing limitations included lack of capacity to identify and mark critical events throughout a data set and to explain additional variables impacting on performance outcomes. The purpose of the work is achieved through the detailing of a novel approach to synthesize EDA data with behavioral data through a two-phase convergent or three-phase complex mixed method design. An example of the implementation of this method would be where EDA and behavior could be monitored during a problem-solving activity to support the identification of elements or stages of a problem that caused increases in cognitive load. This form of investigation could impact on research for instructional or task design. The method could also be employed to evaluate the effect of performance factors such as cognitive abilities or levels of expertise on cognitive load experienced throughout problem solving. Using the method in this manner could contribute towards educational research agendas aiming to develop critical learner competencies through experiences such as problem-orientated learning. Although this method is set in the context of education research, the outlined broad methodological approach of synthesizing EDA and behavioral data sets is envisaged to be relevant and impactful in other areas of social and behavioral science research.

In the context of education, where learners are consistently engaging with tasks and problems, understanding factors relating to performance is essential as experiencing excessive cognitive load can influence both learning and willingness to engage in similar tasks in the future [21,22]. EDA has previously been evaluated for its capacity to effectively measure cognitive load [3]. The presented convergent/complex mixed method advances on the capacity to use EDA to measure cognitive load, and the feasibility of using wearable sensors in educational settings [1], to expand the remit of research questions which can be addressed in both education and cognitive load research. The convergence of EDA data and behavioral observation, supported by video-stimulated recall interviews, allows for further interpretations of the synthesized data to be developed. Collection of such data and gaining understanding of the problem-solving experience in this manner would support moving beyond assumption and speculation towards making clearer inferences about the factors relating to performance. These inferences could support the improvement of the educational experience for individual learners while also contributing to the wider research community by maximizing the use of physiological sensors to enhance the understanding of how pertinent factors relate to problem-solving performance. The approach could also be used as a means of informing curriculum design, specific task design and educational practice.

## Figures and Tables

**Table 1:** Classification of approaches to measuring cognitive load with examples.

	Causal Association	
Objectivity	Direct	Indirect
Subjective	Self-reported difficulty	Self-reported mental effort
Objective	Brain activity	Pupillometry
	Dual-task performance	Electrodermal activity
		Behavioral measures
